# Griffith’s Formulary

**Published:** 1874-04

**Authors:** 


*A Universal Formulary: Containing the methods of Preparing and Administering Officinal and other Medicines.* By Eglesfeld Griffith, M. D. Third Edition, revised and Enlarged. By Jno. M. Maisch, Phar. D. Philadelphia: Henry C. Lea, 1874. Buffalo: T. Butler & Son.

This is also a new Edition of a well known and established work. As a work of reference for druggists and those physicians who prepare their own medicines, it is of much value. Under the editorial supervision of Prof. Maisch, it is made to conform with the present standard of Pharmaceutical science, and will be consulted by those having occasion to use it with profit and pleasure. The additions which have been made to the present edition, embraces a large variety of preparations, and these together with the change in the arrangement have made an almost new volume of it.

